# The effect of remimazolam-based total intravenous anesthesia versus sevoflurane-based inhalation anesthesia on emergence delirium in children undergoing tonsillectomy and adenoidectomy: study protocol for a prospective randomized controlled trial

**DOI:** 10.3389/fphar.2024.1373006

**Published:** 2024-06-25

**Authors:** Hong-Yu Ma, Yu-Hang Cai, John Wei Zhong, Jia Chen, Zhen Wang, Chao-Yi Lin, Qiao-Qiao Wang, Hua-Cheng Liu

**Affiliations:** ^1^ Department of Anesthesiology and Perioperative Medicine, The Second Affiliated Hospital and Yuying Children’s Hospital of Wenzhou Medical University, Wenzhou, Zhejiang, China; ^2^ Key Laboratory of Pediatric Anesthesiology, Ministry of Education, Wenzhou Medical University, Wenzhou, Zhejiang, China; ^3^ Key Laboratory of Anesthesiology of Zhejiang Province, Wenzhou Medical University, Wenzhou, Zhejiang, China; ^4^ Department of Anesthesiology and Pain Management, University of Texas Southwestern Medical Center, Dallas, TX, United States; ^5^ Laboratory Medicine Center, Allergy Center, Department of Transfusion Medicine, Zhejiang Provincial People’s Hospital, Affiliated People’s Hospital, Hangzhou Medical College, Hangzhou, Zhejiang, China

**Keywords:** remimazolam, emergence delirium, sevoflurane, children, general anesthesia

## Abstract

**Background:** Remimazolam, a new ultrashort-acting benzodiazepine, is becoming increasingly applied in general anesthesia. This study is designed to investigate the effect of remimazolam-based total intravenous anesthesia and sevoflurane-based inhalation anesthesia on emergence delirium in pediatric tonsillectomy and adenoidectomy.

**Methods and analysis:** This is a monocentric, prospective, randomized, double-blind clinical trial. A total of 90 pediatric patients will be randomized to receive remimazolam-based total intravenous anesthesia (remimazolam group, n = 45) or sevoflurane-based inhalation anesthesia (sevoflurane group, n = 45). The primary outcome will be the incidence of emergence delirium, which will be evaluated using the Pediatric Anesthesia Emergence Delirium (PAED) scale. The secondary outcomes include the extubation time, recovery time, behavior change using the post-hospitalization behavior questionnaire for ambulatory surgery (PHBQ-AS), and adverse events.

**Ethics and dissemination:** This study has been approved by the Institutional Review Board (IRB) of the Second Affiliated Hospital and Yuying Children’s Hospital of Wenzhou Medical University (2023-K-262-02).

**Clinical trial registration:** ClinicalTrials.gov, identifier NCT06214117.

## Background

Emergence delirium (ED) is defined as a combative, excited, and disoriented behavior that requires transient restraint during emergence from anesthesia. This phenomenon represents a frequent complication in children undergoing general anesthesia, with an incidence from 10% to 20% in general (Malarbi et al., 2011; Doerrfuss et al., 2019). The prevalence of ED is associated with several factors, including the use of volatile anesthetics, type of surgery, age, preoperative anxiety, pre-existing behavior, and patient and parent interaction with healthcare providers (Mason, 2017). In preschool-aged children undergoing tonsillectomy and adenoidectomy with sevoflurane anesthesia, the incidence of emergence delirium (ED) has increased significantly, reaching up to 40%–60% (El-Hamid and Yassin, 2017; Yang et al., 2022).

Although ED in children is mostly self-limited, the presence of ED can not only delay recover and increase healthcare costs but may also decrease parent satisfaction scores with anesthesia (Mason, 2017). Unfortunately, the etiology and pathogenesis of ED is not completely understood, and effective pharmacological interventions to treat this condition are lacking (Tripp et al., 2021; Sobol and Sobol, 2022).

Sevoflurane-based inhalational anesthesia is widely used in pediatric tonsillectomy and adenoidectomy due to its rapid onset, quick emergence, and minimal airway irritation (Yang et al., 2022). However, this approach is accompanied not only with a high incidence of emergence delirium but also with concerns regarding environmental pollution and the exposure of healthcare personnel to inhalational anesthetics (Miyabe-Nishiwaki et al., 2021). The adoption of total intravenous anesthesia (TIVA) can avoid these issues and is becoming a routine in clinical practice (Chandler et al., 2013; Jo et al., 2019). Propofol is the most common intravenous anesthetic drug, preferred for its rapid onset and short duration of action. Previous studies have demonstrated that propofol-based TIVA is associated with a lower incidence of emergence delirium compared to sevoflurane-based inhalational anesthesia (Chandler et al., 2013). Despite its benefits, propofol has various side effects, including pain during injection, addiction, respiratory depression, and propofol-related infusion syndrome (PRIS) (Chen et al., 2022). These drawbacks limit its further application in pediatric patients.

Remimazolam, a new ultrashort-acting benzodiazepine, is becoming increasingly applied in general anesthesia. Compared to propofol, remimazolam exhibits favorable characteristics such as no injection pain, metabolism independent of liver and kidney function, and minimal respiratory depression (Zhao et al., 2023). These attributes make remimazolam particularly well-suited for pediatric patients. The pharmacokinetics and pharmacodynamics of drugs are different in children compared to adults due to the physiological maturation and development of various organs, transporter, and enzyme systems (Rodieux et al., 2022). Despite this, the majority of available research has been conducted in adults, and data on the pediatric population are scarce (Yang et al., 2022; Fang et al., 2023; Gao et al., 2023). Recently, a clinical trial has garnered considerable attention for demonstrating that a single intravenous injection of remimazolam before the end of surgery can significantly reduce emergence delirium in children (Yang et al., 2022). However, research into the efficacy of remimazolam-based TIVA in reducing the incidence of emergence delirium among pediatric patients remains limited. Therefore, this study is designed to compare the occurrence of emergence delirium in children who undergo tonsillectomy and adenoidectomy under remimazolam-based TIVA or sevoflurane-based inhalational anesthesia.

## Materials and methods

### Study design

**FIGURE 1:**
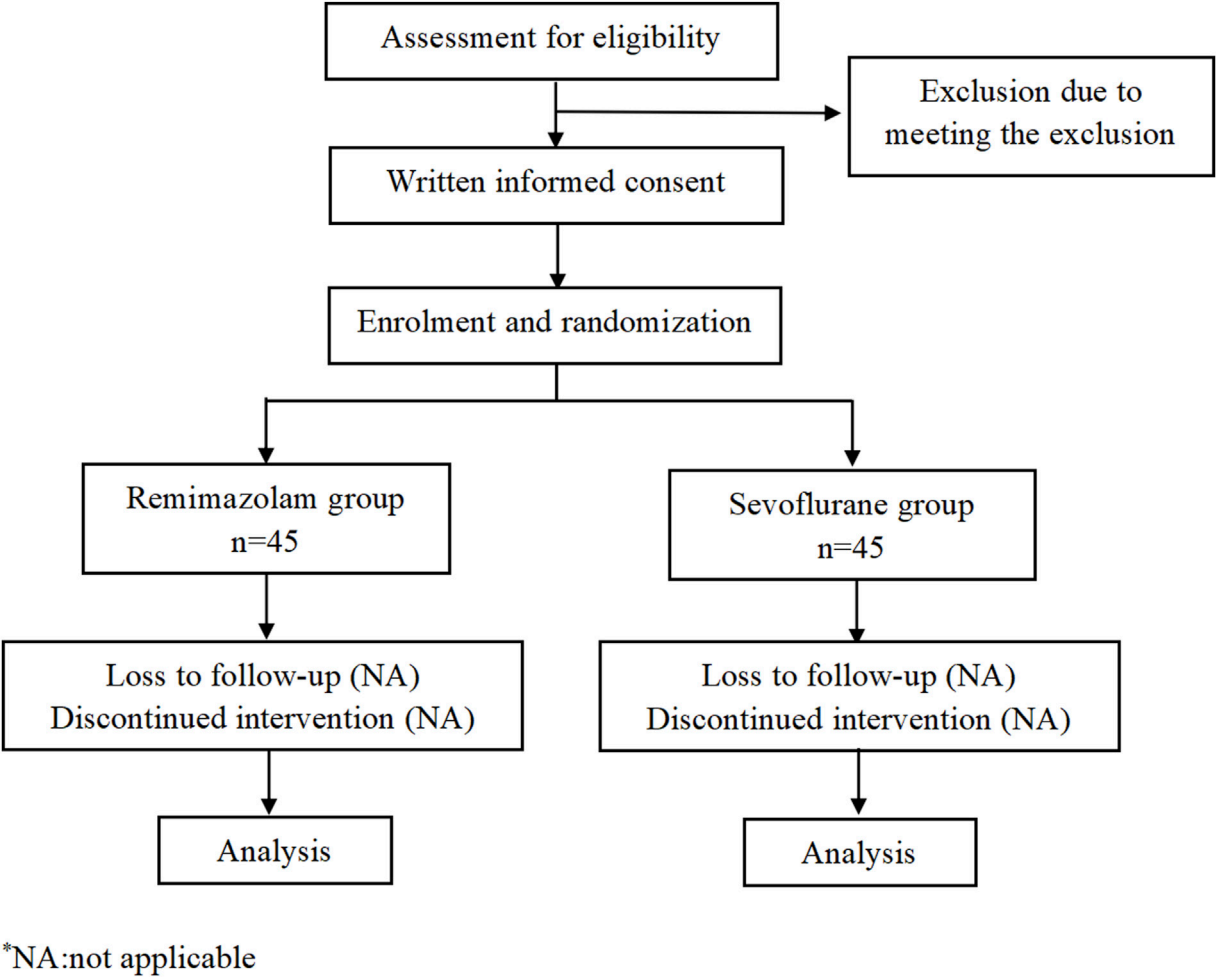
Consort flow diagram.

**FIGURE 2:**
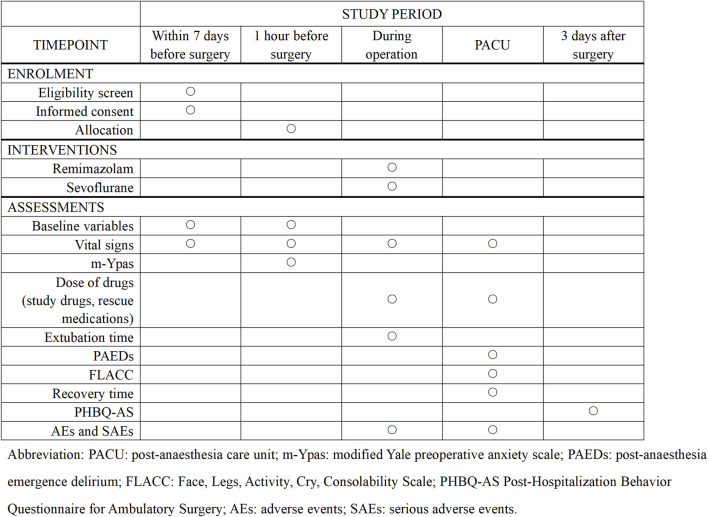
Time frame.

This is a monocentric, prospective, randomized, double-blind study and will be conducted at the Second Affiliated Hospital and Yuying Children’s Hospital of Wenzhou Medical University. A total of 90 pediatric patients will be randomly assigned to two groups: remimazolam group or sevoflurane group. The consort flow diagram and time frame are shown in Figures 1, 2, respectively.

The study protocol has been approved by the Institutional Review Board (IRB) of the Second Affiliated Hospital and Yuying Children’s Hospital of Wenzhou Medical University (2023-K-262-02) and registered at ClinicalTrials.gov (NCT06214117, 18 January 2024). The trial will be conducted in compliance with the study protocol, the Declaration of Helsinki, and good clinical practice (GCP).

### Eligibility criteria

#### Inclusion criteria

1) Age 3–6 years;
2) American Society of Anesthesiologists (ASA) I or II;
3) Booked for tonsillectomy and adenoidectomy;
4) Body mass index (BMI) for age between the 25th and 85th percentiles according to the 2000 Centers for Disease Control and Prevention (CDC) growth charts (Kuczmarski et al., 2002).

#### Exclusion criteria

1) Children (ASA III or IV) who have abnormal liver and kidney function, cardiovascular dysfunction, endocrine dysfunction, or dysfunction of any other organ;
2) Allergy or hypersensitive reaction to remimazolam;
3) Mental disorder;
4) Recent respiratory infection (2 weeks);
5) Other reasons that researchers hold as inappropriate for participation in this trial: under specialized care or living in social welfare institutions or any other factors that could affect their ability to participate in the study.

### Random and binding

Since inclusion and exclusion criteria are carefully designed to limit potential bias, simple randomization will be conducted. Randomization will be performed in a 1:1 ratio according to a computer-generated randomization table (SPSS Inc., Chicago, IL, United States) at the beginning of the study.

The research team will be divided into two independent cohorts: anesthesia implementation researchers and postoperative assessment researchers. Postoperative assessment researchers will not be involved in the implementation and management of anesthesia and be blinded to the study allocation. Unblinding will not be carried out until after the completion of the study when all data will have been entered and the statistical analysis plan will have been confirmed.

### Enrollment

Potential participants will be screened the day before the surgery by an independent researcher. The study protocol, potential risks, potential benefits, and alternatives will be explained to the children and their parents. Moreover, parents will be informed that the data would be analyzed anonymously and that no payment would be received for participation in the study. An independent researcher will be responsible for obtaining written informed consent and for the collection of demographic and baseline data from the participants.

### Intervention

#### Preoperative period

All children will be confirmed to meet the ASA fasting guideline. Demographic and baseline data will be obtained by a member of the research team. No premedication will be given before anesthesia. To mitigate preoperative anxiety and facilitate the completion of venipuncture before anesthesia induction, children will be allowed to watch cartoon videos in the presence of one of their parents or guardians. During the preoperative period, an independent researcher will assess the anxiety level by using the modified Yale Preoperative Anxiety Scale (m-YPAS, Supplementary Table S1).

#### Induction of anesthesia

Upon arriving at the operative area, children will be monitored with continuous electrocardiogram (ECG), heart rate (HR), respiratory rate (RR), pulse oxygen saturation (SpO_2_), and non-invasive blood pressure (NIBP). Those values will be recorded every 5 min.

The children will receive different anesthesia induction regimens based on their group allocation. 1) Remimazolam group: An intravenous administration of fentanyl (Yichang Humanwell Pharmaceutical Co., Ltd., China) at a dosage of 2–3 μg kg^-1^, remimazolam (Yichang Humanwell Pharmaceutical Co., Ltd., China) at 0.3–0.5 mg kg^-1^, and rocuronium (Siegfried Hameln GmbH, Germany) at 0.6 mg kg^-1^ will be given. 2) Sevoflurane group: Anesthesia will be induced with 8% sevoflurane (Maruishi Pharmaceutical Co. Ltd., Osaka, Japan) in 100% oxygen at a flow rate of 6 L min^-1^, followed by intravenous fentanyl at 2–3 μg kg^-1^ and rocuronium at 0.6 mg kg^-1^. Once adequate anesthetic depth is achieved, orotracheal intubation will be performed.

#### Maintenance of anesthesia

After intubation, pressure-controlled mechanical ventilation will be used to maintain the end-tidal carbon dioxide partial pressure (P_et_CO_2_) between 35 and 45 mmHg. 1) In the remimazolam group, remimazolam will be infused at an initial rate of 2 mg kg^-1^. h^-1^ (1–3 mg kg^-1^. h^-1^) and remifentanil (Yichang Humanwell Pharmaceutical Co., Ltd., China) will be administered at an initial infusion rate of 0.25 μg kg^-1^ min^-1^ (0.1–0.5 μg kg^-1^ min^-1^) (Fang et al., 2023). 2) In the sevoflurane group, anesthesia will be maintained at a minimum alveolar concentration (MAC) of 1.0–1.5, with remifentanil being infused at an initial rate of 0.25 μg kg^-1^ min^-1^ (0.1–0.5 μg kg^-1^ min^-1^). Fentanyl will be administered intravenously (0.5–1 μg kg^-1^) as needed when the heart rate exceeds the baseline by more than 20%. All anesthetic medications will be discontinued at the end of the procedure.

Following the induction of anesthesia, an intravenous dose of 0.15 mL kg^-1^ dexamethasone will be administered to prevent postoperative nausea and vomiting (PONV) (Czarnetzki et al., 2008). For postoperative analgesia, an intravenous dose of 1 mg kg^-1^ ketorolac tromethamine will be administered. After completing surgery, neuromuscular blockade will be reversed with neostigmine (0.02 mL kg^-1^) and atropine (0.01 mL kg^-1^). Subsequently, the intubation will be removed when the tidal volume will be ≥ 6 mL kg^-1^ and the frequency ≥ 15.

#### Recovery from anesthesia and follow-up

In the post-anesthesia care unit (PACU), the Pediatric Anesthesia Emergence Delirium (PAED) scale and the Face, Legs, Activity, Cry, and Consolability (FLACC) scale will be used to assess patients at 0, 5, 10, 20, and 30 min after emergence from anesthesia. Patients with pain (FLACC ≥4 and PAED score <10) will be treated with 0.5 μg/kg of fentanyl. In the case of ED (PAED score ≥10 but FLACC <4), initial comfort measures will have been taken. Initial comfort measures include the application of weighted blankets or the provision of gentle and sustained touch by a PACU nurse (Eull et al., 2022; Kim et al., 2024). If these are ineffective, 1 mg kg^-1^ propofol will be administered. If the patient experiences both emergence delirium and pain (PAED score ≥10 and FLACC score ≥4), pain management will be prioritized and then the final PAED score will be re-assessed after 5 min (Somaini et al., 2016). The recovery of the patients from sedation will be assessed after the procedure using the modified Aldrete score (Supplementary Table S2). When the modified Aldrete score reaches 9, the child will be permitted discharge from the PACU.

The behavior change will be scored using the post-hospitalization behavior questionnaire for ambulatory surgery (PHBQ-AS) through a telephone follow-up survey. The study follow-up will be performed at 3 days after surgery in order to avoid the influence of first 2 days’ agitation, significant pain, or nausea on questionnaire response (Lee-Archer et al., 2020).

### Outcomes

#### Primary outcome

The primary outcome will be the incidence of emergence delirium, which will be defined to the Pediatric Anesthesia Emergence Delirium scale (PAED, Supplementary Table S3) ≥10 (Yang et al., 2022). The PAED score will be documented by the same well-trained doctor who will be blinded to the allocation group. The PAED scale has been proved to be a reliable and valid measurement tool for children aged 1–17 years in a post-anesthesia setting (Daoud et al., 2014).

#### Secondary outcomes

The secondary outcomes will include the following:

1) Extubation time: the time interval from the end of the surgery to extubation.
2) Recovery time: the time interval from the end of the surgery to spontaneous eye opening in the PACU.
3) The pain score will be assessed by the Face, Legs, Activity, Cry, and Consolability scale (FLACC, Supplementary Table S4). The FLACC scale has been proven to be a reliable instrument for assessing postoperative pain in children (from birth to 18 years) who have undergone outpatient surgery under general anesthesia (Crellin et al., 2015).
4) The incidence of rescue propofol or fentanyl in the PACU.
5) Behavior change will be assessed by the post-hospitalization behavior questionnaire for ambulatory surgery (PHBQ-AS, Supplementary Table S5) at 3 days. The PHBQ-AS has been validated in children aged 2–7 years (Lee-Archer et al., 2022).
6) The satisfaction scores of the surgery doctor and attending anesthesiologist will be assessed after surgery by a 3-point scale: very satisfied, satisfied, and dissatisfied.
7) Adverse events (AEs):
i. Respiratory adverse events:
  a. Minor adverse events: oxygen desaturation (SpO_2_<90%), airway obstruction, coughing, or wheezing.
  b. Major adverse events: laryngospasm and bronchospasm.
ii. Cardiovascular adverse events: hypotension (SBP <70 mmHg +2 times age) and bradycardia (HR < 70 beats. min^-1^).
iii. Other adverse events: post-operative nausea, vomiting, etc.

### Data collection, analysis, and management

A paper case report form (CRF) will be designed for registration of clinical data and the study result. All data will be meticulously documented in real-time by an independent data recorder. Data will be securely stored in a password-protected computer to safeguard the patients’ confidentiality. The study will adhere closely to the principles of good clinical practice (GCP). A designated investigator will be responsible for collecting, filing, and transferring data, while another independently verifies the accuracy and safety of the information.

For screening failures (patients who were screened but not randomized), a screening failure form will be completed. Investigators must ascertain the reasons for the withdrawal, including withdrawal from study investigations, withdrawal due to adverse events, failure to attend, non-compliance, withdrawal of consent, or other reasons. Participants who withdraw or are withdrawn from the trial will not be replaced. Data on participants after withdrawal will be used and analyzed with the intention-to-treat (ITT) principle.

#### Data monitoring

There are no planned interim analyses for the primary end point, but an independent data and safety monitoring board (DSMB) will regularly review study accrual and unblinded safety data.

#### Safety evaluation

Safety assessments will be composed of monitoring vital signs during the study and observing and recording all adverse events (AEs) and serious adverse events (SAEs).

AEs, defined as all unfavorable/unexpected medical events that occur in patients, whether causally related to the study drugs or not, will be recorded and treated immediately. SAEs are defined as adverse medical events, such as death, life-threatening, permanent, or serious disability or loss of function, and prolonged hospitalization after the subject receives the investigational drug. SAEs will be reported to the Ethics Committee within 24 h. In addition, the researchers will purchase clinical trial insurance, which compensates for treating any harm that occurs during the study.

#### Study discontinuation criteria

This trial will be terminated under the following criteria: 1) clustered serious adverse events are related to intervention measurement with supportive evidence and 2) the administration, including the DSMB, requests that the trial be discontinued.

#### Risks and benefits

There are no additional risks in this study other than the potential risks of standard clinical practice. No participants will receive any direct benefits from the study nor any compensation for their participation.

#### Sample size

The PAED score, which has excellent sensitivity (91%) and specificity (98%), will be used to assess emergence delirium (Janssen et al., 2011). A previous study showed that the rate of emergence delirium in pediatric tonsillectomy and adenoidectomy during sevoflurane anesthesia was approximately 44% (Yang et al., 2022). Hence, a sample size of 72 patients will provide 80% power at a 0.05 level of significance to detect a 30% difference in the proportion of children who attain emergence delirium in each group. Estimating a 20% drop out rate, 90 children will be needed in this study (PASS 15.0).

### Statistical analysis

The statistical analysis will be based on the intention-to-treat principle, including all randomized patients, regardless of subsequent withdrawal or deviation from the protocol. The data will be analyzed by SPSS version 24.0 for Windows (SPSS Inc., Chicago, IL, United States). The normality of distribution of continuous variables will be tested by the one-sample Kolmogorov–Smirnov test. Continuous variables with normal distribution will be presented as the mean ± standard deviation (SD), while non-normal variables will be presented as the median (interquartile range). Continuous variables will be analyzed using the Student’s t-test or Mann–Whitney U test based on the distribution, and categorical variables will be analyzed by the chi-squared test or Fisher’s exact test. Since there is only one primary outcome and the secondary outcomes are considered exploratory, no subgroup analyses or any other additional analyses will be planned in this study. No corrections for multiplicity will be planned. All statistical tests are two-sided, and a *p*-value <0.05 will be considered significant.

### Study duration and trial status

The trial recruitment has begun on 1 April 2024, and the study will end when the final patient has completed their final study visit. The anticipated total study period is 1 year. Upon the completion of the study, data analysis and the preparation of the manuscript are anticipated to take 3 months.

## Discussion

This randomized controlled clinical trial is designed to investigate the effect of remimazolam-based total intravenous anesthesia and sevoflurane-based inhalation anesthesia on emergence delirium in pediatric tonsillectomy and adenoidectomy. The results of the study will help optimize anesthesia techniques for pediatric tonsillectomy and adenoidectomy, thereby improving clinical practices.

Remimazolam is an ultrashort-acting benzodiazepine that acts on GABA receptors, providing a rapid onset and recovery effect. It also has a controllable degree of cardiovascular inhibition and negligible respiratory inhibition (Kilpatrick, 2021). Its efficacy and safety have been demonstrated in the fields of digestive endoscopy, bronchoscopy, hysteroscopy, ICU sedation, general anesthesia, etc. (Pastis et al., 2019; Tang et al., 2022; Zhao et al., 2023). In terms of sedative efficacy, remimazolam is superior to midazolam and non-inferior to propofol (Pastis et al., 2019; Ko et al., 2023). In a pharmacokinetic study performed in pediatric patients, intravenous infusion remimazolam showed good controllability with a high clearance of 15.9 (12.9, 18.2) mL kg^-1^ min^-1^ (median, Q25, Q75) and a short terminal half-life of 67 (49, 85) min (Gao et al., 2023). Considering its favorable pharmacodynamics and pharmacokinetics, remimazolam may emerge as an optimal choice for intravenous anesthesia in pediatric patients.

The pharmacokinetics (PK) and pharmacodynamics (PD) of drugs are different in children compared to adults (Welzel et al., 2022). Children have a larger amount of extracellular fluid and total body water compared to adults, which may be due to the larger volume of drug distribution (Murat et al., 1996). In addition, inter-individual variability in pharmacokinetics (PK) and pharmacodynamics (PD) in the pediatric population is typically due to age and body size. Hence, appropriate doses used for adults are often not appropriate for children. Since the clinical research on remimazolam in pediatric patients is limited, the dosage of remimazolam in this study is based on our previous research results (Fang et al., 2023). The doses of other drugs in this study are based on previously published studies in children (Sammartino et al., 2010; Chen et al., 2024).

The association of benzodiazepines with the occurrence of emergence delirium remains to be discussed. Nevertheless, recent studies have reported that remimazolam is associated with a decreased incidence of emergence delirium (Duan et al., 2023; Yang et al., 2023). A study conducted by Duan et al. found that remimazolam not only effectively reduced the incidence of ED compared with propofol but also demonstrated minor hemodynamic effects and a lower occurrence of postoperative adverse events in geriatric hip-replacement patients (Duan et al., 2023). Yang et al. demonstrated that older adult patients undergoing general anesthesia with remimazolam had a low incidence of emergence delirium, similar to propofol (Yang et al., 2023). In the pediatric population, a study found that a single bolus of remimazolam 0.2 mg kg^-1^ at the end of the surgery resulted in a significantly lower incidence of emergence delirium (from 44% to 12%) after sevoflurane anesthesia (Yang et al., 2022).

This study will control for and assess confounders of ED in children, such as the type of surgery, age, patient anxiety, pre-existing behaviors, and interactions with healthcare providers (Mason, 2017). Although the assessment of preoperative anxiety levels has often been overlooked in previous studies, it is noteworthy that in pediatric patients with preoperative anxiety, the reported incidence of postoperative delirium was 3.5 times higher than in non-anxious individuals (Choi et al., 2022). This study will utilize m-YPAS for evaluating preoperative anxiety levels in children. In addition, to address the challenge of blinding due to different anesthesia methods during surgery, we will ensure the integrity of blinding efficacy by having postoperative assessors who are not involved in intraoperative management.

The diagnosis of emergence delirium (ED) in pediatric patients presents a significant challenge, frequently confounded by the presence of postoperative pain (Somaini et al., 2016). To address this issue, our study will alleviate postoperative pain through the administration of opioid and non-steroidal analgesic agents during surgery. Furthermore, it is crucial to perform distinct assessments of emergence delirium and postoperative pain levels (Sikich and Lerman, 2004). For individuals experiencing both postoperative pain and delirium, pain relief is administered prior to delirium assessment, minimizing the impact of postoperative pain on the diagnosis of emergence delirium (Somaini et al., 2016). In the data analysis phase, we will compare pain severity by the FLACC score and the total postoperative analgesic consumption between the two groups. This will provide a more objective evaluation.

In addition, total intravenous anesthesia offers several advantages, such as a lower incidence of respiratory complications and postoperative nausea and vomiting (Kovac, 2021; Karam et al., 2023). Respiratory complications during anesthesia recovery are particularly crucial in pediatric otolaryngologic surgeries (Shen et al., 2022). This study also expects to explore whether remimazolam may contribute to a reduction in this complication. However, this study is a single-center clinical investigation. If the findings of this study yield positive results, we plan to conduct a multicenter clinical trial to further validate the aforementioned hypothesis.

## Strengths and limitations

The main strength of the study is the novelty of the work and the rigorous methodological design. If the result of this RCT is found positive, it will provide a new anesthetic regimen that may reduce emergence delirium and environmental pollution. Another strength of the study is that the sample size was sufficiently powered to detect a clinically important difference. However, there are some limitations to this study. First, there is no alternative to inhalational anesthesia in the control group. Second, it is a single-center trial; multicenter trials will be required in the future.

## Abbreviations

AEs Adverse events; ASA American Society of Anesthesiologists; BMI Body mass index; CRF Case report form; CDC Centers for Disease Control and Prevention; CI Confidence interval; DSMB Data and safety monitoring board; ECG Electrocardiogram; ED Emergence delirium; P_et_CO_2_ End-tidal carbon dioxide partial pressure; FLACC Face, Legs, Activity, Cry, and Consolability; GCP Good clinical practice; HR Heart rate; IRB Institutional Review Board; ITT Intention-to-treat; MAC Minimum alveolar concentration; m-Ypas Modified Yale Preoperative Anxiety Scale; NIBP Non-invasive blood pressure; PAED Pediatric Anesthesia Emergence Delirium; PACU Post-anesthesia care unit; PHBQ-AS Post-hospitalization behavior questionnaire for ambulatory surgery; PONV Postoperative nausea and vomiting; PRIS Propofol-related infusion syndrome; SpO_2_ Pulse oxygen saturation; RR Respiratory rate; SAEs Serious adverse events; SD Standard deviation; TIVA Total intravenous anesthesia.
