# Supplementary material for: The effect of remimazolam-based total intravenous anesthesia versus sevoflurane-based inhalation anesthesia on emergence delirium in children undergoing tonsillectomy and adenoidectomy: study protocol for a prospective randomized controlled trial
Source: Front Pharmacol. 2024 Jun 25;15:1373006. doi: 10.3389/fphar.2024.1373006 (PMC11231196; doi:10.3389/fphar.2024.1373006)
Supplement: Supplementary file 1 [file Table1.docx]

**Table S1** The modified Yale Preoperative Anxiety Scale

| categories | items | scores |
| --- | --- | --- |
| Activity | Looking around, curious, playing with toys, reading (or other age-appropriate behavior); moves around holding area/treatment room to get toys or to go to parent; may move toward operating room equipment | 1 |
|  | Not exploring or playing, may look down, fidget with hands, or suck thumb (blanket); may sit close to parent while waiting, or play has a definite manic quality | 2 |
|  | Moving from toy to parent in unfocused manner, non-activity-derived movements; frenetic/frenzied movement or play; squirming, moving on table; may push mask away or cling to parent | 3 |
|  | Actively trying to get away, pushes with feet and arms, may move whole body; in waiting room, running around unfocused, not looking at toys, will not separate from parent, desperate clinging | 4 |
| Vocalizations | Reading (nonvocalizing appropriate to activity), asking questions, making comments, babbling, laughing, readily answers questions but may be generally quiet; child too young to talk in social situations or too engrossed in play to respond | 1 |
|  | Responding to adults but whispers, “baby talk,” only head nodding | 2 |
|  | Quiet, no sounds or responses to adults | 3 |
|  | Whimpering, moaning, groaning, silently crying | 4 |
|  | Crying or may be screaming “no” | 5 |
|  | Crying, screaming loudly, sustained (audible through mask) | 6 |
| Emotional expressivity | Manifestly happy, smiling, or concentrating on play | 1 |
|  | Neutral, no visible expression on face | 2 |
|  | Worried (sad) to frightened, sad, worried, or tearful eyes | 3 |
|  | Distressed, crying, extreme upset, may have wide eyes | 4 |
| State of apparent arousal | Alert, looks around occasionally, notices or watches what anesthesiologist does (could be relaxed) | 1 |
|  | Withdrawn, sitting still and quiet, may be sucking on thumb or have face turned into adult | 2 |
|  | Vigilant, looking quickly all around, may startle to sounds, eyes wide, body tense | 3 |
|  | Panicked whimpering, may be crying or pushing others away, turns away | 4 |
| Use of parents | Busy playing, sitting idle, or engaged in age-appropriate behavior and doesn’t need parent; may interact with parent if parent initiates the interaction | 1 |
|  | Reaches out to parent (approaches parent and speaks to otherwise silent parent), seeks and accepts comfort, may lean against parent | 2 |
|  | Looks to parent quietly, apparently watches actions, doesn’t seek contact or comfort, accepts it if offered or clings to parent | 3 |
|  | Keeps parent at distance or may actively with-draw from parent, may push parent away or desperately clinging to parent and not let parent go | 4 |

**Table S2** the modified Aldrete score

| Categories | items | scores |
| --- | --- | --- |
| Activity | Able to move 4 extremities voluntarily or on command | 2 |
|  | Able to move 2 extremities voluntarily or on command | 1 |
|  | Unable to move extremities voluntarily or on command | 0 |
| Respiration | Able to breathe deeply and cough freely | 2 |
|  | Dyspnea or limited breathing | 1 |
|  | Apneic | 0 |
| Circulation | BP ± 20% of pre-anesthetic level | 2 |
|  | BP ± 20% to 49% of pre-anesthetic level | 1 |
|  | BP ± 50% of pre-anesthetic level | 0 |
| Consciousness | Fully awake | 2 |
|  | Arousable on calling | 1 |
|  | Not responding | 0 |
| O₂ Saturation | Able to maintain O₂ saturation >92% on room air | 2 |
|  | Needs O₂ inhalation to maintain O₂ saturation >90% | 1 |
|  | O₂ saturation <90% even with O₂ supplement | 0 |

**Table S3** Pediatric anesthesia emergence delirium (PAED) scale

| Behavior | | Not at all | Just a little | Quite a bit | Very much | Extremely |
| --- | --- | --- | --- | --- | --- | --- |
| Makes eye contact | | 4 | 3 | 2 | 1 | 0 |
| Actions are purposeful | 4 | 3 | 2 | 1 | 0 |  |
| Aware of surroundings | 4 | 3 | 2 | 1 | 0 |  |
| Restless | 0 | 1 | 2 | 3 | 4 |  |
| Inconsolable | 0 | 1 | 2 | 3 | 4 |  |

1—Calm; 2—not calm but could be easily consoled; 3—moderately agitated or restless and not easily calmed; 4—combative, excited, thrashing around.

**Table S4** Face, Legs, Activity, Cry and Consolability (FLACC) scale

| categories | items | Score |
| --- | --- | --- |
| Face | No particular expression or smile | 0 |
|  | Occasional grimace or frown; withdrawn, disinterested | 1 |
|  | Frequent to constant frown, clenched jaw, quivering chin | 2 |
| Legs | Normal position or relaxed | 0 |
|  | Uneasy, restless, tense | 1 |
|  | Kicking or legs drawn up | 2 |
| Activity | Lying quietly, normal position, moves easily | 0 |
|  | Squirming, shifting back and forth, tense | 1 |
|  | Arched, rigid, or jerking | 2 |
| Cry | No cry (awake or asleep) | 0 |
|  | Moans or whimpers, occasional complaint | 1 |
|  | Crying steadily, screams or sobs; frequent complaints | 2 |
| Consolability | Content, relaxed | 0 |
|  | Reassured by occasional touching, hugging, or being talked to; distractable | 1 |
|  | Difficult to console or comfort | 2 |

Interpreting the total Behavioral Score: 0 relaxed and comfortable, 1–3 mild discomfort, 4–6 moderate pain, 7–10 severe discomfort or pain or both.

**Table S5** Post Hospitalization Behavior Questionnaire for Ambulatory Surgery（PHBQ-AS）

| 1. Does your child make a fuss about eating?  2. Does your child spend time just sitting or lying and doing nothing?  3. Is your child uninterested in what goes on around him (or her)?  4. Does your child get upset when you leave him (or her) alone for a few minutes?  5. Does your child need a lot of help doing things?  6. Is it difficult to get your child interested in doing things (like playing games with toys)?  7. Does your child have temper tantrums?  8. Is it difficult to get your child to talk to you?  9. Does your child have bad dreams at night or wake up and cry?  10. Does your child have trouble getting to sleep at night?  11. Does your child have a poor appetite? |
| --- |

Each item is scored as follows: 1 much less than before, 2 less than before, 3 same as before / not applicable, 4 more than before, 5 much more than before.
